# Heat and mass transfer analysis of s-PTT nanofluid in microchannels under combined electroosmotic and pressure-driven flows with wall slip using the homotopy perturbation method

**DOI:** 10.1016/j.heliyon.2024.e39526

**Published:** 2024-10-20

**Authors:** Mahtiam Kananipour, Mehdi Moayed Mohseni, Reza Jahanmardi, Hossein Ali Khonakdar

**Affiliations:** a Department of Chemical Engineering, Science and Research Branch, Islamic Azad University, Tehran, Iran; b Department of Polymer Processing, Iran Polymer and Petrochemical Institute, Tehran, Iran

**Keywords:** Electroosmotic flow, Viscoelastic nanofluids, s-PTT model, HPM solution method, Slip condition, Brownian motion

## Abstract

The heat and mass transfer of the electroosmotic flow in microchannel transporting viscoelastic nanofluid is investigated considering Brownian motion of nanoparticles and slip boundary conditions. The simplified Phan-Thien-Tanner model is employed to describe the rheological behavior of fluid and the nonlinear Navier model with non-zero slip critical shear stress is considered at walls. The governing nonlinear momentum, mass, and heat transfer equations are solved using the Homotopy Perturbation Method. The study reveals that increasing the fluid elasticity, nanoparticle concentration, and size significantly enhances the flow rate, heat and mass transfer. Additionally, elasticity and Reynolds number decrease the friction factor. Reducing the double-layer thickness and increasing the Reynolds number lead to higher flow rates and fluid velocities. Notably, the findings emphasize the critical role of the slip conditions on the Sherwood and Nusselt numbers.

## 1 Introduction

Studying how liquids flow through microchannels under the influence of electric fields fascinates scientists worldwide. The survey of electroosmotic flow in microchannels has always provided valuable insights into this phenomenon and its many possible applications. Electroosmotic forces drive liquids through tiny channels using electricity. The reason for this is the charged walls of the channel, which attract oppositely charged particles in the liquid so that they move in the desired direction. The movement of ions in the microchannel creates fluid motion, a phenomenon known as electroosmotic flow. Electroosmotic transport uses an electric field to move fluids through narrow channels, like those in microchips, to improve flow speed and efficiency beyond natural forces such as gravity [1,2]. These advantages include the elimination of mechanical parts, simple and precise control of flow by changing the electric field, improved mixing of fluids due to a velocity gradient along the microchannel, the ability to generate pulsed and modulated flows, and the reduction of interfacial effects and pressure drop within the microchannel. Researchers have explored many applications of electroosmotic flow, including drug delivery, biosensors, separation of molecules, synthesis of nanoparticles, and power generation [3]. Numerous studies have focused on how microchannel designs, fluid properties, boundary conditions, and external forces affect electroosmotic transport [4].

The Phan-Thien–Tanner model is the most widely used non-linear viscoelastic constitutive model derived from network theory [5,6]. Among the advantages of this model compared to other non-Newtonian fluids, in addition to the ability to provide analytical solutions, the following can be mentioned: 1- Ability to predict the rheological behavior of the viscoelastic fluid. 2- Diagnosis of‏ ‏the shear thinning behavior of the fluid. 3- Capability to accurately predict elongational viscosity.

Many fluids used in microchannels and microfluidics exhibit non-Newtonian properties, particularly viscoelasticity. Nanofluids are fluids containing small amounts of nanoparticles, usually with a nano-dimension less than 100 nm, made from metals, metal oxides, or carbon-based materials, suspended in a base fluid [7]. Due to the importance of the topic, many researches have been conducted in the field of nanofluid which the following can be mentioned. Investigating the effect of micro-organisms along with nanofluid [8], the entropy generation in systems containing nanofluid [9], hybrid nanofluids [10] and the use of artificial neural networks in nanofluids [11,12].

The characteristics of nanofluids highly desirable for various thermal and mechanical applications, including heat transfer enhancement, energy efficiency improvement, friction reduction, and energy consumption minimization. Selecting specific nanoparticles allows for tailored nanofluid properties, further expanding their potential applications [[13], [14], [15]]. Brownian motion, the random movement of nanoparticles in the base fluid due to collisions with fluid molecules, affects the thermophysical properties of the nanofluid by causing uniform distribution and preventing settling. This movement increases the heat transfer between the nanoparticles and the base fluid [16]. The Buongiorno model is often used to model Brownian motion in nanofluids. In this context, we consider nanoparticles as a phase of the base fluid, considering effects such as sliding, thermophoresis, diffusion, and the density of the nanoparticles [17]. Nanofluids exhibit unique properties, including enhanced heat and mass transfer as well as improved electrical conductivity. These properties enable researchers to develop more sustainable and efficient energy conversion technologies [18]. In addition, it is essential to study the electroosmotic flow in nanofluids in filtration and separation processes. When combined with the electroosmotic flow, nanoparticles are more advanced in removing pollutants, particles, and ions than the base fluid, providing significant solutions for water treatment, wastewater management, and treatment systems. Studying the interactions between the components of the nanofluid and the impregnated surfaces is critical to ensure acceptable and stable performance. In addition, understanding the effects of factors such as pH, temperature, and nanoparticle concentration on electroosmotic flow behavior in nanofluids provides valuable insights for system design and optimization [19].

Magesh et al. [20] examined the influence and effects of electroosmotic flow on the dynamics and behavior of a Jeffrey fluid flowing through microchannels that exhibit structural and compositional heterogeneity. This research the employed Poisson-Boltzmann equations to investigate the double layer, determine the fluid velocity, and perform a linear analysis of the stream function within the framework of a low Reynolds number approximation by considering the relevant variables.

Wang et al. [21] examined a swirling electroosmotic flow within a parallel plate microchannel characterized by high zeta potential. The researchers utilized the Navier slip law for the boundaries and calculated the electric double-layer potential distribution through the nonlinear Poisson-Boltzmann equation. The study conducted a thorough investigation encompassing a detailed examination of the effects of slip length and wall zeta potential on the velocity distribution. Shim et al. [22] conducted a comprehensive investigation into microfluidic systems, focusing on an in-depth analysis of parameters related to diffusion phoresies and diffusion osmosis, as well as their effects on these systems. They also examined the complex interplay between these phenomena. Using the PTT model, an approach widely used in scientific research, Sarma et al. [23] investigated to analyze the phenomenon of electroosmotic flow in parallel plate microchannels, with special focus on viscoelastic fluids that exhibit high zeta potential. They hypothesized that the fluid network connections demonstrate effective slip motion instead of continuous motion. Liang et al. [24] examined the influence of slip boundary conditions on swirling electroosmotic flow. The study showed that slip can reduce the boundary stress effect and enhance flow development, indicating that the non-Newtonian fluid parameter and slip coefficient play a significant role in swirling electroosmotic flow. An augmentation in the non-Newtonian parameter decelerates the flow and diminishes the velocity magnitude. Squires et al. [4] elucidated the fundamental principles and applications of microfluidics, encompassing electroosmotic flow in nanofluids. The discussion delved into the physics dictating fluid dynamics at the nanoliter scale and emphasized the significance of electroosmotic flow within microchannels. Jean et al. [25] conducted experimental investigations on convective heat transfer phenomena and flow characteristics of nanofluids. They conducted experiments using aqueous nanofluids comprising nanoparticles of varying concentrations and sizes. The findings indicate that nanofluids exhibit a notable improvement in heat transfer efficiency compared to base fluids, with the extent of enhancement correlating with the concentration and dimensions of the nanoparticles. The study also indicated that the convective heat transfer coefficient increased as the Reynolds numbers and nanoparticle concentrations increased. Des et al. [26] studied nanoparticle impact on nanofluid thermal conductivity, considering concentrations, sizes, and temperature changes. Nanofluid thermal conductivity factors include nanoparticle amount, size, and temperature, offering insights into their behavior at different temperatures. Wen et al. [27] studied nanofluids to improve heat transfer at the onset of flow and found that nanoparticles, influenced by concentration and size, enhance heat transfer over the base fluid, providing insights into nanofluid dynamics. Siva et al. [28] presented an analysis of the heat transfer of the combined magnetohydrodynamic (MHD) and electroosmotic flow (EOF) of non-Newtonian fluid in a rotating microchannel. The authors have obtained an exact solution for the energy transport equation and used it to determine the flow velocity and volume flow rates under appropriate boundary conditions. The effects of various parameters, such as the rotational Reynolds number, Joule heating parameter, couple stress parameter, Hartmann number, and buoyancy parameter, on the flow velocities and temperature are examined in detail.

This study aims to answer this question. What is the synchronous effect of Brownian motion of the nanofluid, the viscoelastic rheology of base fluid, the slip condition at walls, the influence of critical shear stress on slip condition, the simultaneous imposition of two pressure and electroosmotic driving forces and the effects of high zeta potential on the flow, thermal and concentration characteristics of nanofluid in microchannel. Therefore, the s-PTT model as the viscoelastic base fluid and the nonlinear Navier slip model with non-zero critical shear stress as the slip boundary condition was chosen. ‏ ‏ So far, no research has been reported in the literature that has investigated these factors simultaneously, and therefore their effect on the system is still unknown.

The Homotopy Perturbation Method (HPM) is used to efficiently solve the momentum, mass, and heat transport equations, as it is particularly suitable for treating nonlinear systems. HPM is an effective approach for tackling non-linear problems, integrating traditional perturbation and homotopy methods to address their individual shortcomings. A key advantage of HPM is its independence from a small parameter, allowing its results to remain valid across a wider range of values. In contrast, other methods often rely on such parameters, where an inappropriate selection can lead to significant errors [29,30]. Additionally, determining the right parameter in many non-linear equations can be challenging. However, one notable drawback of semi-analytical methods is the complexity of the resulting large equations.

**Table:** Nomenclature

C	specific heat at constant pressure	$\left[\frac{J}{Kg.k}\right]$	sh	local Sherwood number	
K	Debye-Hückel parameter	[$\frac{1}{m}]$	T	Temperature	[k]
$C_{0}$	nanoparticle volume fraction at the microchannel inlet		$T_{0}$	temperature at the microchannel inlet	[k]
$C_{w}$	nanoparticle volume fraction at the microchannel wall		$T_{w}$	temperature on the microchannel wall surface	[k]
$D_{B}$	Brownian diffusion coefficient	$\left[\frac{m^{2}}{s}\right]$	u	fluid velocity	[$\frac{m}{s}$]
$D_{T}$	thermophoretic diffusion coefficient	$\left[\frac{m^{2}}{s}\right]$	$u_{m}$	the average velocity of the fluid	[$\frac{m}{s}$]
E	charge of a proton	[ c]	$u_{sh}$	Helmholtz-Smoluchowski velocity	[$\frac{m}{s}$]
$E_{s}$	streaming potential	[v]	$u_{w}$	wall velocity	[$\frac{m}{s}$]
Μ	dynamic viscosity of the fluid	$\left[\frac{kg}{m.s}\right]$	x,y	Cartesian coordinates	[m]
H	half distance between the upper and lower microchannel walls	[m]	$N_{b}$	Brownian motion parameter	
L	length of the microchannel	[m]	$N_{t}$	thermophoresis parameter	
$n_{i}$	ionic number concentration of the ith species		*Nu*	local Nusselt number	
P	Pressure	[Pa]	$n_{0}$	the bulk ionic concentration of type i ions	$\left[\frac{1}{m^{3}}\right]$
Kb	Boltzmann constant	[$\frac{J}{mol.k}$]	Z	ionic capacity	
*Kn*	Knudsen number	[$\frac{J}{k}$]	F	electrical body force	[$\frac{N}{m^{3}}$]
$C_{f}$	local skin friction coefficients	[$\frac{kg}{m^{3}}$]	Θ	dimensionless temperature distribution	
Ψ	electric potential	[v]	Φ	the dimensionless nanoparticle volume fraction	
Ζ	zeta potential	[v]	$\rho_{e}$	charge density	[$\frac{c}{m^{3}}]$
Γ	Dimensionless pressure		$\tau_{w}$	wall stress	
Α	nanofluid thermal dispersion	[$\frac{m^{2}}{s}]$	$\tau_{xy}$	shear stress	
ε	the dielectric constant of the medium		$\dot{\gamma }$	velocity gradient	
$\varepsilon_{0}$	vacuum permittivity	$\left[\frac{c}{v.m}\right]$	Τ	the ratio of the heat capacity of the nanoparticle to that of the fluid	
$\lambda_{0}$	Fuid, electrical conductivity	$\left[\frac{1}{\Omega .m}\right]$	$\rho_{f}$	fluid density	$\left[\frac{kg}{m^{3}}\right]$
					

## 2 Mathematical formulation for other fields

### 2.1 Governing equation

The current problem investigates a hydrodynamically fully developed, laminar, steady, and incompressible flow of a viscoelastic fluid, examining the effects on nanofluid movement, heat, and mass transfer in a microchannel with parallel plates. Fig. 1a and 1b provide visual representations. The horizontal axis aligns with the channel walls, and the vertical axis is perpendicular. The study uses the center of the microchannel as the reference point for the coordinate system, where H stands for half the height of the channel, L denotes its length, and W signifies its width. The study simplified the geometry of the problem by assuming the width is significantly smaller than the height, allowing it to model the problem as a two-dimensional nonlinear microchannel flow involving electric double-layer (EDL) phenomena. The continuity equation and the momentum equations govern the flow dynamics in this transport phenomenon. These equations are fundamental to understanding the phenomenon and form the basis for analyzing the electroosmotic flow in microchannels with parallel plates. Eq. (1) is continuity equation for an incompressible fluid as follows:

(1) ∇.u=0

**Fig. 1a:**
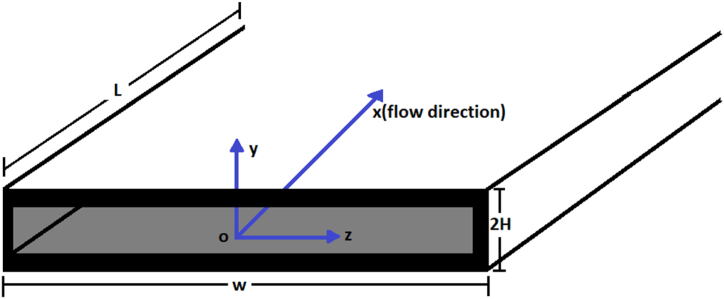
Schematic of problem-3D.

**Fig. 1b:**
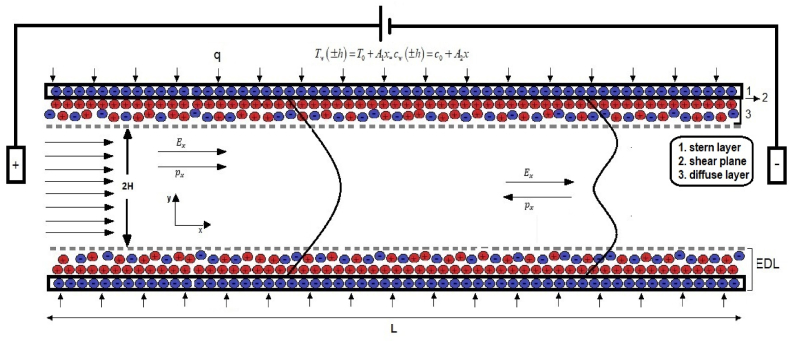
Schematic of problem-2D.

The momentum equation, once modified, can be expressed as:

(2) $$\rho \frac{Du}{Dt}=\nabla .\tau -\nabla P+F$$

Where u is the velocity vector, P is the pressure, t is the time, ρ is the fluid density, and τ is the polymeric extra stress contribution. Eq. (3)provides the body force, denoted as F in Eq. (2) [31].:

(3)(a) $$F=\rho_{e}E$$

(3)(b) E=−∇ϕ

E is the applied external electric field, ϕ is the electric potential, and $\rho_{e}$ is the net electric charge density in the fluid.

### 2.2 Analytical solution to the electrostatic potential

Based on the electrostatic theory, the Poisson equation describes the relationship between the electric potential ψ and the charge density $\rho_{e}$ near a flat surface [32]:

(4) $$\nabla^{2}\psi =-\frac{\rho_{e}}{\epsilon }$$

where ψ denotes the EDL potential and ϵ is the dielectric constant of the solution.

Assuming a constant dielectric coefficient and negligible fluctuations, the ion distribution in a symmetric electrolyte solution follows the equilibrium Boltzmann distribution which is shown by Eq. (5) [33]:

(5) n=in0exp(−zeψkBT)

The bulk ionic concentration is denoted by $n_{i}$ and z represents the valence of the ions. E is the charge of a proton, $k_{B}$ is the Boltzmann constant, and T is the absolute temperature.

The net charge density in a unit volume of the fluid is given by Ref. [34]:

(6) $$\rho_{e}=\left(n_{+}-n_{-}\right)ze=-2n_{0}ze\;\sinh\left(-\frac{ze\psi }{k_{B}T}\right)$$

By substituting Eq. (6) into the Poisson Eq. (4), the Poisson-Boltzmann equation becomes [35]:

(7) $$\frac{d^{2}\psi }{dy^{2}}=\frac{2n_{0}ze}{\epsilon }\sinh\left[\frac{ze}{k_{B}T}\psi \right]$$

The relevant boundary conditions for Eq. (7) are given by Eq. (8):

(8) $$\psi \left(\pm h\right)=\bar{\zeta }=\frac{ze\zeta }{k_{B}T}$$

To obtain the dimensionless form of Eq. (7), we introduce the following dimensionless variables pertinent to this analysis.

$$y=\bar{y}h,k=\frac{\bar{k}}{h},\bar{\psi }\left(y\right)=\frac{ze\psi }{k_{B}T}=\frac{\bar{\zeta }}{\zeta }\psi ,\bar{\rho }\left(y\right)=\frac{\rho_{e}}{n_{0}ze}$$

The dimensionless form of Eq. (7) is obtained as Eq. (9):

(9) $$\frac{d^{2}\bar{\psi }\left(\bar{y}\right)}{d{\bar{y}}^{2}}={\bar{k}}^{2}\;\mathrm{Sinh}\left[\bar{\psi }\left(\bar{y}\right)\right]$$

In the equation studied the Hückel discharge parameter ($k^{2}=\left(2e^{2}z^{2}n_{0}\right)/\left(k_{B}T\epsilon \right)$)shows an inverse relationship with the layer thickness, affecting the flow rate. This parameter is essential in analyzing the electroosmotic flow in parallel plate microchannels. The electric double layer (EDL) thickness is assumed to be much smaller than the channel height, meaning the charge distribution in the EDL remains constant when fluid velocity is low or the EDL is thin enough. The flow pattern becomes symmetric and fully developed, thereby making it possible to disregard the kinetic energy component in the flow equation. When potential values are minimal, the Poisson-Boltzmann equation can be simplified using the approximation (Sinh ψ ≈ ψ (indicating that the ion's electric potential energy is significantly lower than its thermal energy. Thus, a simplified Poisson-Boltzmann equation (Eq. 10) applies to channel flow [36].

(10) $$\frac{d^{2}\bar{\psi }\left(\bar{y}\right)}{d{\bar{y}}^{2}}={\bar{k}}^{2}\bar{\psi }\left(\bar{y}\right)$$

Which has the analytical solution:

(11) $$\bar{\psi }\left(\bar{y}\right)=\frac{\left(e^{\bar{k}\left(1-\bar{y}\right)}+e^{\bar{k}\left(1+\bar{y}\right)}\right)\bar{\zeta }}{1+e^{2\bar{k}}}$$

Exponential functions often make equations complex. Our study overcomes this issue by employing Taylor series expansion for simplification and solving equations.

(12) $$\exp\left[\bar{k}\;\bar{y}\right]=1+k\;y+\frac{k^{2}y^{2}}{2}+\frac{k^{3}y^{3}}{6}+\frac{k^{4}y^{4}}{24}+\frac{k^{5}y^{5}}{120}+\frac{k^{6}y^{6}}{720}+\frac{k^{7}y^{7}}{5040}+O{\left[y\right]}^{8}$$

In this equation, O [y] is the truncation error.

To facilitate the solution, Eq. (11) is derived first and then expanded using Eq. (12), which shows by Eq. (13).

(13) $$\frac{d\bar{\psi }\left(\bar{y}\right)}{d\bar{y}}=\frac{\left(-e^{\bar{k}\left(1-\bar{y}\right)}\bar{k}+e^{\bar{k}\left(1+\bar{y}\right)}\bar{k}\right)\bar{\zeta }}{1+e^{2\bar{k}}}=B_{1}\left(B_{2}\bar{y}+B_{3}{\bar{y}}^{3}+B_{4}{\bar{y}}^{5}+B_{5}{\bar{y}}^{7}\right)$$

Where the appendix elucidates the coefficients B.

### 2.3 Constitutive equations for simplified Phan-Thien–Tanner (s-PTT) model

The rheological behavior of the viscoelastic fluid was determined using the simplified Phan-Thien–Tanner (s-PTT) model. The s-PTT constitutive equations, in their general form, are [5,23,37]:

(14) $$f\left(\tau_{kk}\right)\tau +\lambda \tau^{\nabla }=2\eta D$$

(15) $$D=\frac{1}{2}\left(\nabla u+\nabla u^{T}\right)$$

D is the rate of deformation tensor, λ is the relaxation time, η is the viscosity coefficient, $\tau^{\nabla }$ is the derivative of the stress tensor, and τ is the stress tensor, which is defined as follows:

(16) $$\tau^{\nabla }=\frac{D\tau }{Dt}-\nabla u^{T}.\tau -\tau .\nabla u$$

The stress coefficient function is expressed in a linear form as:

(17) $$f\left(\tau_{kk}\right)=1+\frac{\varepsilon \lambda }{\eta }\tau_{kk}$$

The expansibility parameter ε controls the level of nonlinearity in the s-PTT model, which describes the behavior of viscoelastic fluids. As ε approaches zero, the model simplifies to the upper-convected Maxwell model, suitable for dilute polymer solutions. By imposing a velocity condition and utilizing a set of Eq. (14), (15), (16), (17)), the study derives a set of equations for further analysis or calculations [38].

(18)(a) $$f\left(\tau_{kk}\right)\tau_{xx}=2\lambda \gamma ˙\tau_{xy}$$

(18)(b) $$f\left(\tau_{kk}\right)\tau_{xy}=\eta \gamma ˙+\lambda \gamma ˙\tau_{yy}$$

(18)(c) $$f\left(\tau_{kk}\right)\tau_{yy}=0$$

By setting $\tau_{yy}$ to zero and combining the two equations Eq. 18a and Eq. (18b), we can derive the following relation:

(19) $$\tau_{xx}=\frac{2\lambda {\tau_{xy}}^{2}}{\eta }$$

According to the obtained equation and Eq. (2), the equation for the fully developed channel flow becomes:

(20) $$\frac{d\tau_{xy}}{dy}=-\rho_{e}E_{x}+p_{x}$$

The pressure gradient ($p_{x})$, the electric field ($E_{x})$, and the electric potential of the external field ($\rho_{e})$ assume symmetry at the channel walls and no overlap of the EDL. Therefore, the shear stress ($\tau_{xy})$ and the electric potential (ψ) vanishes at the y = 0 plane. Using Eq. (4), Eq. (20) can be combined to yield the following expression:

(21) $$\tau_{xy}=\epsilon \frac{d\psi }{dy}E_{x}+p_{x}y+\gamma_{1}$$

It defined the shear-rate asymmetry coefficient as $\gamma_{1}=\tau_{1}/\eta$ for simplicity. The constant $\tau_{1}$, which represents shear stress, will be determined later using boundary conditions. It's important to note that this coefficient doesn't directly correspond to physical property.

Using Eqs. (18), (19) and (21) together, the following expression (Eq. (22)) for the velocity gradient was derived:

(22) $$\gamma ˙=\frac{\tau_{xy}}{\eta }\left(1+\frac{2\epsilon \lambda^{2}}{\eta^{2}}{\left(\tau_{xy}\right)}^{2}\right)$$

The presented equations can be non-dimensionalized using similarity variables:

$$\bar{\psi }=\frac{\bar{\zeta }}{\zeta }\psi ,\bar{\tau }=\frac{\tau \;h}{\eta \;u_{sh}},\bar{\gamma }˙=\frac{\dot{\gamma }\;h}{u_{sh}},u_{sh}=\frac{-\varepsilon \;\zeta \;E_{x}}{h},\bar{u}=\frac{u}{u_{sh}},p_{x}=-\frac{\Gamma \;E_{x}\;\varepsilon \;\zeta }{h^{2}}=\frac{\Gamma \eta \;u_{sh}}{h^{2}},De=\lambda \;u_{sh}k$$

The dimensionless form of Eqs. (23), (24)) are as follows:

(23) $$\bar{\tau_{xy}}=-\frac{1}{\bar{\zeta }}\frac{d\bar{\psi }}{d\bar{y}}+\Gamma \;\bar{y}+{\bar{\gamma }}_{1}$$

(24) $$\bar{\gamma }˙=\frac{du}{dy}={\bar{\tau }}_{xy}\left(1+\frac{2\epsilon De^{2}}{{\bar{k}}^{2}}{\left({\bar{\tau }}_{xy}\right)}^{2}\right)$$

### 2.4 Analytical solution for velocity distribution

#### 2.4.1 Slip at the walls of the channel

The nonlinear Navier slip law (Eq. (25)) is an experimental model that relates the slip velocity and the shear stress at the wall by a power law function as follows [39,40]:

(25) $$u_{w}={\left(\pm \frac{\tau_{w}}{\beta }\right)}^{\frac{1}{s}}$$

In this equation, s denotes the power index. Analysis of the slip condition shows that slip occurs when the shear stress exceeds a critical value ($\tau_{c}$). After applying the slip condition, the final form of the power law equation can be expressed as follows:

(26) $$\left\{ \begin{array}{l} u_{w}=0\;\pm \tau_{w}\leq \tau_{c}\\ u_{w}={\left(\frac{\pm \tau_{w}-\tau_{c}}{\beta }\right)}^{\frac{1}{s}}\;\pm \tau_{w}\rangle \tau_{c} \end{array}\right.$$

By making Eq. (26) dimensionless, we obtain Eq. (27).

(27) $$\left\{ \begin{array}{l} u_{w}=0\;\pm \tau_{w}\leq \text{Bc}\\ u_{w}={\left(\frac{\pm \tau_{w}-\text{Bc}}{B}\right)}^{\frac{1}{s}}\;\pm \tau_{w}\rangle \text{Bc} \end{array}\right.$$

The non-dimensional variables are defined as follows, where B is the dimensionless slip number, and Bc is the dimensionless slip critical shear stress number:

$$\tau_{w}=\tau_{\text{xy}}\left(+1\right),B=\frac{\beta {\text{hu}}^{s-1}}{\eta_{0}},\text{Bc}=\frac{\tau_{c}h}{\eta_{0}u}$$

By integrating Eq. (24) and imposing the slip boundary condition ($u\left(\pm 1\right)=u_{w}$), the velocity profile is obtained inform Eq. (28).

(28) $$\bar{u}=c_{21}+\frac{B_{6}y^{2}}{2}+\frac{B_{12}y^{4}}{4}+\frac{B_{13}y^{6}}{6}+\frac{B_{14}y^{8}}{8}+\frac{B_{15}y^{10}}{10}+\frac{B_{16}y^{12}}{12}+\frac{B_{17}y^{14}}{14}+\frac{B_{18}y^{16}}{16}+\frac{B_{19}y^{18}}{18}-\frac{B_{10}y^{20}}{20}-\frac{B_{11}y^{22}}{22}$$

For this problem, the non-dimensional volumetric flow rate)Q (, is given by Eq. (29):

(29) $$\bar{Q}={\bar{u}}_{avg}=\frac{1}{2h}\int_{-h}^{+h}\bar{u}d\bar{y}=\frac{1}{h}\int_{0}^{+h}\bar{u}d\bar{y}$$

### 2.5 Heat and mass transfer equations

The governing equations which represent the conservation of the total mass, thermal energy, and the nanoparticle volumetric fraction are expressed by Eqs. (30), (31)).

(30) $$\left(u.\nabla \right)T=\alpha \nabla^{2}T+\tau \left[D_{B}\nabla T.\nabla C+\left(\frac{D_{T}}{T_{0}}\right)\nabla T.\nabla T\right]+\frac{1}{\rho c}\varphi$$

(31) $$\left(u.\nabla \right)C=D_{B}\nabla^{2}C+\left(\frac{D_{T}}{T_{0}}\right)\nabla^{2}T$$

φ is the viscous dissipation term, defined by Eq. (32).

(32) $$\varphi =\tau_{xy}\left(\frac{\partial u_{x}}{\partial y}\right)$$

The equation above has the following parameters: the diffusion coefficient of Brownian motion ($D_{B}$), the thermal diffusion coefficient ($D_{T}$), the nanofluid thermal diffusion coefficient (α), the inlet temperature of the microchannel ($T_{0}$), and the ratio of the heat capacity of the nanoparticle to that of the fluid (τ).

For flow in a parallel channel, the researchers assume that only one velocity component is nonzero, meaning that the fluid particles move in the same direction. If only the u component of the velocity is nonzero and v is zero everywhere, then the continuity equation implies that u is independent of x, as ∂u/∂x=0. Similarly, the hydraulic pressure (p) only depends on the fluid motion, so it is a function of x only, and the pressure gradient ∂p/∂x is constant.

Furthermore, we assume the temperature (T) and the nanoparticle volumetric fraction (C) on both walls change linearly with x, such that:

$$T_{w}\left(x\right)=T_{0}+A_{1}x,C_{w}\left(x\right)=C_{0}+A_{2}x$$

$T_{0}$ and $C_{0}$ are the reference temperature and nanoparticle volumetric fraction at the channel entrance, respectively; since T and C vary linearly with x, we have $\partial^{2}T/\partial x^{2}=\partial^{2}C/\partial x^{2}=0$ [41]. These assumptions simplify the continuity and other governing equations. The simplified of Eqs. (30), (31)) are expanded by Eqs. (33), (34)).

(33) $$u\frac{\partial T}{\partial x}=\alpha \frac{\partial^{2}T}{\partial y^{2}}+\tau D_{B}\left(\frac{\partial T}{\partial x}\frac{\partial C}{\partial x}+\frac{\partial T}{\partial y}\frac{\partial C}{\partial y}\right)+\frac{\tau D_{T}}{T_{0}}\left({\left(\frac{\partial T}{\partial x}\right)}^{2}+{\left(\frac{\partial T}{\partial y}\right)}^{2}\right)+\frac{\tau_{xy}}{\rho c}\frac{\partial u}{\partial y}$$

(34) $$u\frac{\partial C}{\partial x}=D_{B}\frac{\partial^{2}C}{\partial y^{2}}+\frac{D_{T}}{T_{0}}\frac{\partial^{2}T}{\partial y^{2}}$$

By making the temperature and mass distributions dimensionless (Eqs. (35), (36))) and applying the boundary conditions (Eq. (37)), dimensionless temperature and mass distributions are derived.

(35) $$\theta \left(y\right)=\frac{T-T_{w}}{A_{1}h},\phi \left(y\right)=\frac{c-c_{w}}{A_{2}h}$$

(36) θ(±1)=0

(37) ϕ(±1)=0

(38) $$\theta^{\prime \prime }+Nb\left(1+\theta '\phi '\right)+Nt\left(1+{\left(\theta^{\prime }\right)}^{2}\right)+{\left(\frac{1}{u_{m}}\right)}^{2}\;\mathrm{Pr}\times Ec\times \tau_{xy}\times \bar{u}\prime -\frac{1}{u_{m}}\text{Re}\times \mathrm{Pr}\times u=0$$

(39) $$\phi^{\prime \prime }+\frac{Nt}{Nb}\theta^{\prime \prime }-\frac{u}{u_{m}}\mathrm{Pr}\times \text{Re}\times Le=0$$

The non-dimensional variables are defined as follows:

$$Nb=\frac{\tau D_{B}\left(A_{2}\right)h}{\alpha },Nt=\frac{\tau D_{T}\left(A_{1}\right)h}{\alpha T_{0}},\mathrm{Pr}=\frac{\nu }{\alpha },\text{Re}=\frac{u_{m}h}{\nu },Ec=\frac{{u_{m}}^{2}}{cA_{1}h},Le=\frac{\alpha }{D_{B}},u_{m}=\frac{1}{2h}\int_{-h}^{+h}u\left(y\right)dy=\frac{1}{h}\int_{0}^{+h}u\left(y\right)dy$$

In this study, the main variables of interest are the local skin friction, Nusselt number, and Sherwood number which are shown by Eq. (40) a-c. These quantities are computed only on the lower wall due to the symmetrical nature of the flow. The expression is as follows:

(40)(a) $$C_{f}=\frac{\tau_{w}}{\rho_{f}{u_{m}}^{2}}$$

$$\tau_{w}={\left(\tau_{xy}\right)}_{y=-h}$$

(40)(b) $$Nu=\frac{xq_{wT}}{k_{f}\left(T_{w}-T_{o}\right)}$$

$$q_{wT}=-k_{f}{\left(\frac{\partial T}{\partial y}\right)}_{y=-h}$$

(40)(c) $$Sh=\frac{xq_{wc}}{D_{B}\left(C_{w}-C_{o}\right)}$$

$$q_{wc}=-D_{B}{\left(\frac{\partial C}{\partial y}\right)}_{y=-h}$$

The dimensionless form of the above equations are obtained inform Eq. (41) a-c:

(41)(a) $$Nu=-\theta^{\prime }\left(-1\right)$$

(41)(b) $$Sh=-\phi^{\prime }\left(-1\right)$$

(41)(c) $$C_{f}=\frac{\bar{\tau }}{\text{Re}u_{m}}\left(-1\right)$$

### 2.6 HPM solution and discussion

Conventional methods cannot solve the highly complex Eq. (38) and Eq. (39). To address the challenges in finding analytical solutions to such problems, due to the absence of an analytical solution, we use an approximate method. To explain the basis of this process, consider the following nonlinear differential equation and boundary condition [42,43].

(42) A(u)−q(r)=0,r∈Ω

$$B\left(u,\frac{\partial u}{\partial n}\right)=0,r\in \Gamma$$

Where q(r) is an analytic function, B is a boundary operator, Γ is the boundary of the domain Ω and A(u) is a differential operator consisting of two parts: linear L(u) and nonlinear N(u). Thus, Eq. (42) can be rewritten as Eq. (43).

(43) L(u)+N(u)−q(r)=0

The perturbation function is expressed by Eq. (44):

(44) $$H\left(w,p\right)=\left(1-p\right)\left[L\left(w\right)-L\left(u_{0}\right)\right]+p\left[N\left(w\right)-q\left(r\right)\right]$$

When p is set 0 and 1, Eq. (44) becomes to Eq. (45) a and b.

(45)(a) $$H\left(w,0\right)=L\left(w\right)-L\left(u_{0}\right)=0$$

(45)(b) H(w,1)=A(w)−q(r)=0

The approximation solution of Eq. (44) could be expressed as Eq. (46).

(46) $$w=w_{0}+pw_{1}+p^{2}w_{2}+\ldots$$

If p set to one the approximation solution of problem is obtained as Eq.47

(47) $$f=\lim_{p\to 1}w=w_{0}+w_{1}+w_{2}+\ldots$$

Eq. (38) and Eq. (39) can be expanded based on the homotopy construction as Eqs. (48) and (49).

(48)(a) $$H1=\left(\left(1-p\right)\left(L_{1}\left(\theta \right)-L_{1}\left(\theta_{0}\right)\right)\right)+p\left(L_{1}\left(\theta \right)+\left(Nb\left(1+\theta '\phi '\right)+Nt\left(1+{\left(\theta^{\prime \prime }\right)}^{2}\right)+\left({\left(\frac{1}{u_{m}}\right)}^{2}\left(\mathrm{Pr}\times Ec\times u^{\prime }\times \tau_{xy}\right)\right)-\left(\frac{\text{Re}\times \mathrm{Pr}\times u}{u_{m}}\right)\right)\right)$$

(48)(b) $$H2=\left(\left(1-p\right)\left(L_{2}\left(\phi \right)-L_{2}\left(\phi_{0}\right)\right)\right)+p\left(L_{2}\left(\phi \right)+\frac{Nt}{Nb}\theta^{\prime \prime }-\frac{\text{Re}\times \mathrm{Pr}\times Le\times u}{u_{m}}\right)$$

(49)(a) $$\theta =\theta_{0}\left[y\right]+p\theta_{1}\left[y\right]+p^{2}\theta_{2}\left[y\right]+p^{3}\theta_{3}\left[y\right]+p^{4}\theta_{4}\left[y\right]+\ldots$$

(49)(b) $$\phi =\phi_{0}\left[y\right]+p\phi_{1}\left[y\right]+p^{2}\phi_{2}\left[y\right]+p^{3}\phi_{3}\left[y\right]+p^{4}\phi_{4}\left[y\right]+\ldots$$

The solution continued up to 4 steps which are presented in Eqs (50), (51), (52), (53), (54), (55), (56), (57), (58), (59), (60)).

(50) $$\theta_{0}=0$$

(51) θ1=c3+B20y22+B22y412+B23y630+B24y856+B34y1090+B26y12132+B27y14182+B28y16240+B29y18306+B30y20380+B31y22462+B32y24552+B33y26650+B25y28756−By3021870

(52) $$\theta_{2}=0$$

(53) θ3=c11+B50y412+B51y630+B52y856+B53y1090+B62y12132+B64y14182+B65y16240+B66y18306+B76y20380+B68y22462+B69y24552+B70y26650+B71y28756+B72y30870+B73y32992+B74y341122+B75y361260+B67y381406+B63y401560+B56y421722+B57y441892+B58y462070+By48592256+B60y502450+B61y522652+B55y542862+B54y563080+B48y583306−B49y603540

(54) θ4=c15+B77y412+B78y630+B79y856+B80y1090+B81y12132+B82y14182+B83y16240+B84y18306+B85y20380+B86y22462+B87y24552+B88y26650+B89y28756+B90y30870+B91y32992+B92y341122+B93y361260+B94y381406+B95y401560+By42961722+B97y441892+B98y462070+B99y482256+B100y502450+B101y522652+B102y542862+B103y563080−B104y583306+B105y603540

(55) $$\phi_{0}=0$$

(56) $$\phi_{1}=c_{4}+\frac{B_{35}y^{2}}{2}+\frac{B_{36}y^{4}}{12}+\frac{B_{37}y^{6}}{30}+\frac{B_{38}y^{8}}{56}+\frac{B_{39}y^{10}}{90}+\frac{B_{40}y^{12}}{132}+\frac{B_{41}y^{14}}{182}+\frac{B_{42}y^{16}}{240}+\frac{B_{43}y^{18}}{306}+\frac{B_{44}y^{20}}{380}-\frac{B_{45}y^{22}}{462}-\frac{B_{46}y^{24}}{552}$$

(57) $$\phi_{2}=c_{8}-\frac{Nt}{Nb}\left(\begin{array}{l} \frac{B_{20}y^{2}}{2}+\frac{B_{22}y^{4}}{12}+\frac{B_{23}y^{6}}{30}+\frac{B_{24}y^{8}}{56}+\frac{B_{34}y^{10}}{90}+\frac{B_{26}y^{12}}{132}+\frac{B_{27}y^{14}}{182}+\\ \frac{B_{28}y^{16}}{240}+\frac{B_{29}y^{18}}{306}+\frac{B_{30}y^{20}}{380}+\frac{B_{31}y^{22}}{462}+\frac{B_{32}y^{24}}{552}+\frac{B_{33}y^{26}}{650}+\frac{B_{25}y^{28}}{756}-\frac{B_{21}y^{30}}{870} \end{array}\right)$$

(58) $$\phi_{3}=0$$

(59) $$\begin{array}{l} \phi_{4}=c_{16}-\frac{Nt}{Nb}(\frac{B_{50}y^{4}}{12}+\frac{B_{51}y^{6}}{30}+\frac{B_{52}y^{8}}{56}+\frac{B_{53}y^{10}}{90}+\frac{B_{62}y^{12}}{132}+\frac{B_{64}y^{14}}{182}+\frac{B_{65}y^{16}}{240}+\frac{B_{66}y^{18}}{306}+\frac{B_{76}y^{20}}{380}+\\ \frac{B_{68}y^{22}}{462}+\frac{B_{69}y^{24}}{552}+\frac{B_{70}y^{26}}{650}+\frac{B_{71}y^{28}}{756}+\frac{B_{72}y^{30}}{870}+\frac{B_{73}y^{32}}{992}+\frac{B_{74}y^{34}}{1122}+\frac{B_{75}y^{36}}{1260}+\frac{B_{67}y^{38}}{1406}+\frac{B_{63}y^{40}}{1560}+\frac{B_{56}y^{42}}{1722}+\\ \frac{B_{57}y^{44}}{1892}+\frac{B_{58}y^{46}}{2070}+\frac{B_{59}y^{48}}{2256}+\frac{B_{60}y^{50}}{2450}+\frac{B_{61}y^{52}}{2652}+\frac{B_{55}y^{54}}{2862}+\frac{B_{54}y^{56}}{3080}+\frac{B_{48}y^{58}}{3306}-\frac{B_{49}y^{60}}{3540}) \end{array}$$

(60) $$\begin{array}{l} \phi_{5}=c_{20}-\frac{Nt}{Nb}(\frac{B_{77}y^{4}}{12}+\frac{B_{78}y^{6}}{30}+\frac{B_{79}y^{8}}{56}+\frac{B_{80}y^{10}}{90}+\frac{B_{81}y^{12}}{132}+\frac{B_{82}y^{14}}{182}+\frac{B_{83}y^{16}}{240}+\frac{B_{84}y^{18}}{306}+\\ \frac{B_{85}y^{20}}{380}+\frac{B_{86}y^{22}}{462}+\frac{B_{87}y^{24}}{552}+\frac{B_{88}y^{26}}{650}+\frac{B_{89}y^{28}}{756}+\frac{B_{90}y^{30}}{870}+\frac{B_{91}y^{32}}{992}+\frac{B_{92}y^{34}}{1122}+\frac{B_{93}y^{36}}{1260}+\frac{B_{94}y^{38}}{1406}+\\ \frac{B_{95}y^{40}}{1560}+\frac{B_{96}y^{42}}{1722}+\frac{B_{97}y^{44}}{1892}+\frac{B_{98}y^{46}}{2070}+\frac{B_{99}y^{48}}{2256}+\frac{B_{100}y^{50}}{2450}+\frac{B_{101}y^{52}}{2652}+\frac{B_{102}y^{54}}{2862}+\frac{B_{103}y^{56}}{3080}-\frac{B_{104}y^{58}}{3306}+\frac{B_{105}y^{60}}{3540}) \end{array}$$

According to Eq. (47), the final solution can be obtained by Eqs. (61), (62)).

(61) $$\theta =\theta_{0}+\theta_{1}+\theta_{2}+\theta_{3}+\theta_{4}$$

(62) $$\phi =\phi_{0}+\phi_{1}+\phi_{2}+\phi_{3}+\phi_{4}+\phi_{5}$$

## 3 Results and discussion

Due to the symmetry induced by the boundary conditions, the analysis focuses solely on the upper half of the microchannel, ranging from (y = 0 to y = +1). A comparison with the numerical results obtained from Mathematica software solver is made for the dimensionless temperature and concentration profiles that are shown in Fig. 2-a and 2-b. As it is seen, there is high compliance between results of two solutions.

**Fig. 2a:**
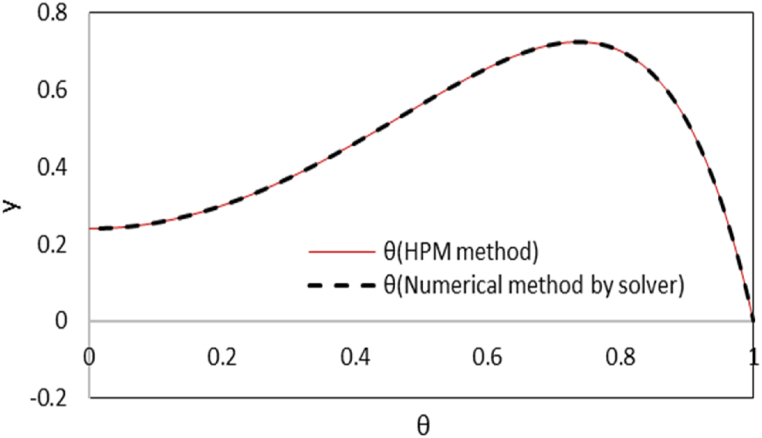
Comparison of dimensionless temperature distribution between HPM and numerical method by Mathematica software at Γ = −1, ${\varepsilon De}^{2}$ = 0.3, S = ζ = *Le* = B = 1, Ec = Pr = 5, Bc = Nb=Nt = 0.1, k = 3 and Re = 0.5.

**Fig. 2b:**
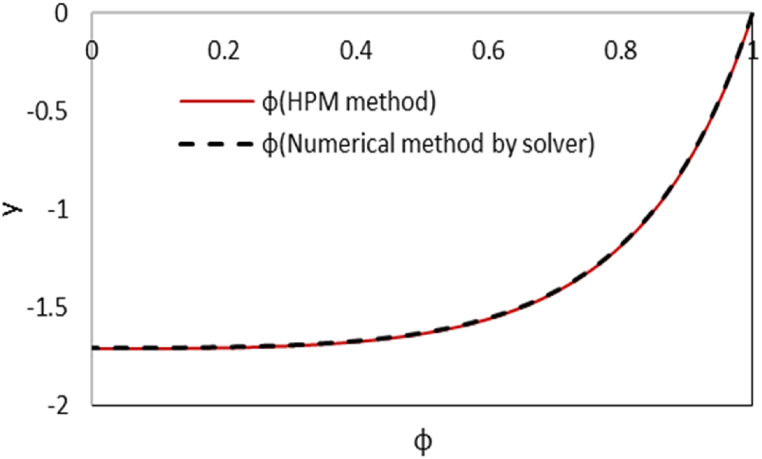
Comparison of nanoparticles distribution between HPM and numerical method by Mathematica software at Γ = −1, ${\varepsilon De}^{2}$ = 0.3, S = ζ = *Le* = B = 1, Ec = Pr = 5, Bc = Nb=Nt = 0.1, k = 3 and Re = 0.5.

**Fig. 3:**
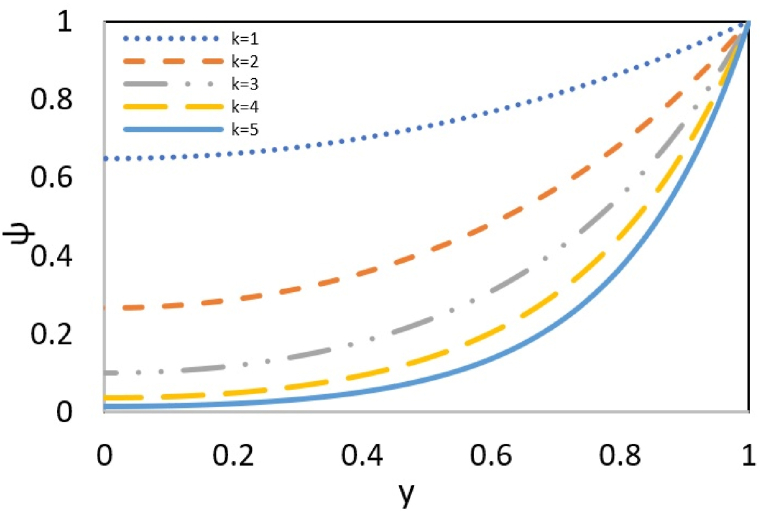
Effect of k parameter on ψ profile at ζ = 1.

The analysis in Fig. 3 looks at how the thickness of the electric double layer affects the surface potential. The boundary conditions ensure a uniform convergence of all the diagrams to a stable value for the streaming potential distribution at the channel wall (ζ = 1).

Since k represents the inverse of the double-layer thickness, an increase in k indicates a decrease in the double layer thickness. When the double layer thickness decreases, fewer ions accumulate near the wall, and more ions will be in the bulk fluid. As a result, the input electric potential decreases as it moves from the wall toward the channel centerline. Consequently, there is a sharper drop in electric potential. Therefore, the surface potential decreases as the double layer thickness decreases and k increases.

**Fig. 4:**
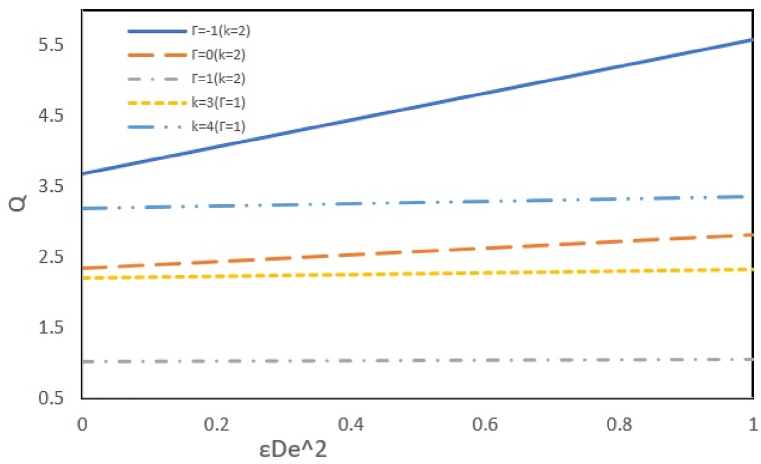
Effect of k and Γ on volumetric flow rate Q versus ${\varepsilon De}^{2}$ at ζ = B = s = 1 and Bc = 0.1.

The flow rate depends on k, elasticity, and slip parameters. An increase in the $\varepsilon De^{2}$ signifies heightened elasticity resulting from the shear-thinning behavior of the fluid. The heightened elasticity causes a reduction in the fluid flow resistance and increases the flow rate, as depicted in Fig. 4. As mentioned earlier, increasing the parameter k enhances the potential driving force for the flow motion, resulting in an increased flow rate. Additionally, this figure investigates the effect of the pressure parameter on the flow rate, as this parameter can act on the fluid in two ways: the same or opposite direction to the electroosmotic flow. When the two forces act on the fluid in the same direction (-Γ), they reinforce each other's effects and increase the flow rate. The impacts of electroosmotic flow and external pressure weaken each other when they act in opposite directions (+Γ), the electroosmotic flow pushing the fluid forward. In contrast, the pressure is applied in the opposite direction, resulting in a decreasing the flow rate.

Fig. 5(a)investigates the effects of two parameters, pressure, and the thickness of the double layer, on the velocity profile. When two forces act upon the fluid in the same direction, the fluid velocity increases with higher values of the parameters k and Γ, reaching its maximum value at the channel center. When two opposing forces interact, an increase in pressure can occur while the layer thickness remains constant, intensifying the interaction between the forces. This allows the flow to continue as long as the electroosmotic force can drive the fluid, as illustrated in the figure. However, by assuming that the pressure effect opposes the electroosmotic flow, an increase in pressure weakens the driving impact of the electroosmotic force, such that at a specific pressure gradient, these two forces completely neutralize each other and, as a result, the fluid flow stops. As the pressure continues to increase, a reverse flow can occur. Additionally, a decrease in k results in an increase in the thickness of the electric double layer and a subsequent reduction in the induction of electric current into the fluid, leading to an increased effect of pressure with distance from the wall, causing a reverse flow. Fig. 5(b) examines how slip parameters at the wall affect the velocity profile. The slip condition reflects the discrepancy between the force applied to the wall and the response of the fluid to this force. Therefore, slip condition at the wall reduces flow resistance and heightens maximum velocity.

**Fig. 5a:**
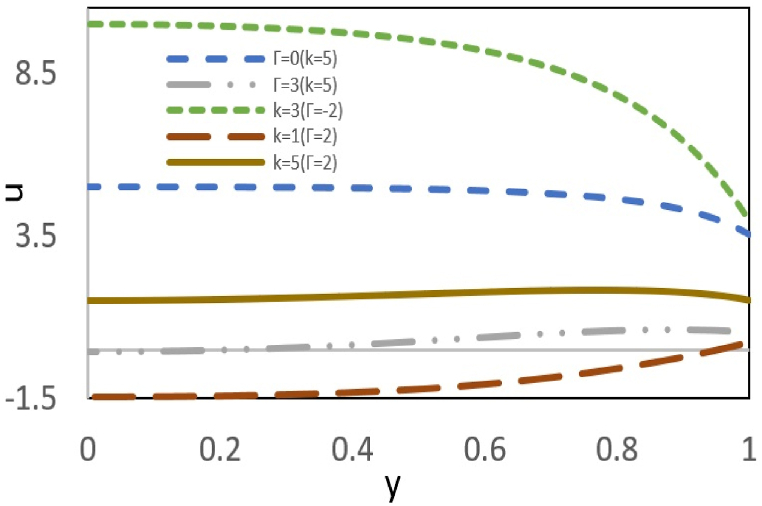
Effect of Γ, k on the velocity profile at ζ = B=Bc = s = 1 and ${\varepsilon De}^{2}$ = 1.

**Fig. 5b:**
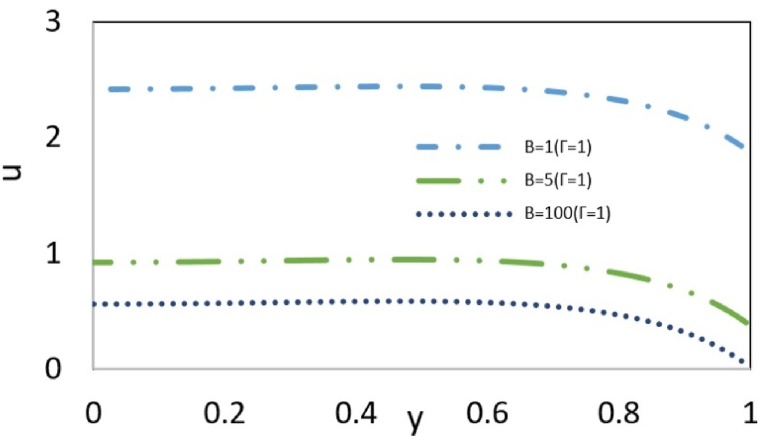
Effect of slip condition and Deborah number on the velocity profile at Bc = ζ = s = Γ = 1, k = 4 and ${\varepsilon De}^{2}$ = 1.

For validation purposes, the velocity equation derived in this study is compared to the velocity equation developed by Sarma et al. [23] Due to the differences in how stress and velocity gradient are nondimensionalized in these two studies, one should compare the velocity ratio to average velocity. Their study was done on an s-PTT viscoelastic fluid under electroosmotic flow in conditions without pressure forces and slip critical shear stress. Therefore, it is expected that by choosing minimal values for pressure gradient and slip critical shear stress number, the velocity profile obtained for this study is close to the velocity profile obtained in their research. This way, one can verify the accuracy of the obtained results for a viscoelastic nanofluid. As illustrated inFig. 5(c), there is high compliance between the results of the two solutions, which substantiates the accuracy of the equations obtained.

**Fig. 5c:**
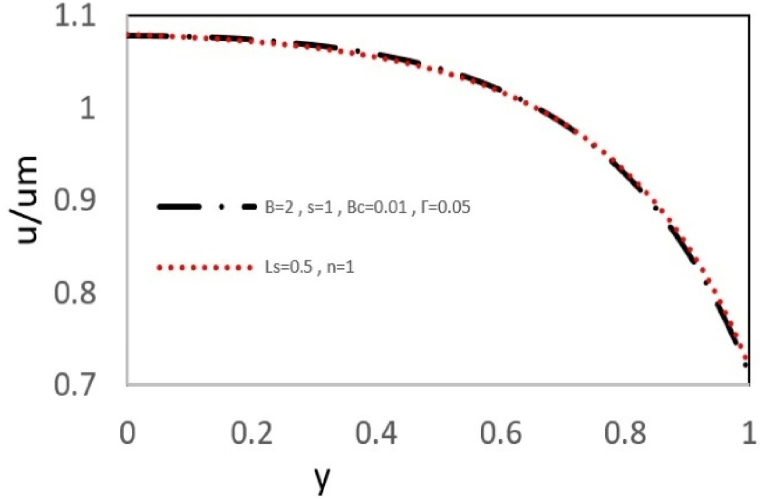
Comparison of normalized velocity (u/um) with Sarma et al. [23] at ζ = 1, k = 4 and ${\varepsilon De}^{2}$ = 1. Black lines: Analytical solutions given by Eq. (28); Red lines: Results of Sarma et al. [23].

**Fig. 6:**
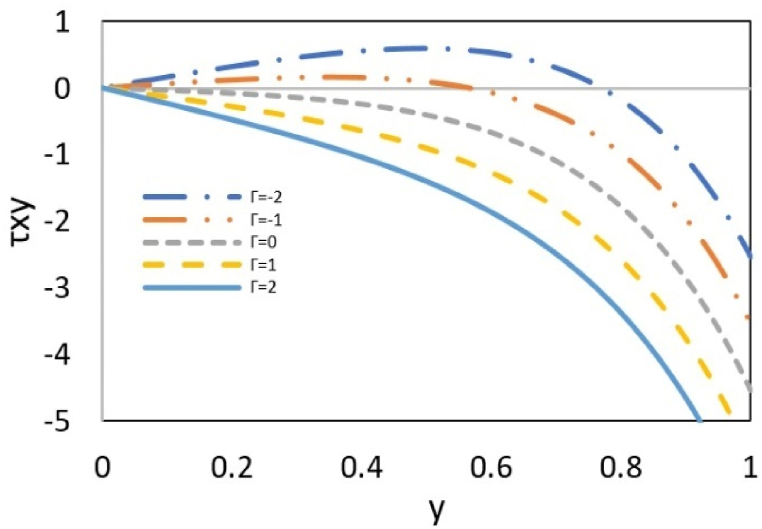
Effect of Γ on shear stress at k = 5, ${\varepsilon De}^{2}$ = 1 and ζ = 1.

In Fig. 6, considering the thickness of the double layer to be constant, the effect of the pressure parameter on the stress has been investigated. The interaction between pressure forces and electroosmotic flow creates a synergistic effect for negative pressure gradient, which enhances flow velocity, and stress levels. Conversely, when these forces act in opposition, there is a noticeable decrease in both the flow rate and stress level. Additionally, stress levels diminish as the electroosmotic effect weakens with increasing distance from the wall. In the case of a positive pressure gradient, where pressure and electroosmotic forces act on the fluid in opposing directions, the stress profile exhibits a change in sign. Since pressure is an isotropic quantity, it maintains a constant value across the cross-section. However, electroosmotic forces are more potent near the wall, diminishing their influence as the distance from the wall increases. Hence, in the space near the wall, due to the dominance of the electroosmotic force, the flow direction is the same as the electroosmotic force, and shear stress is negative, but by moving away from the wall, the pressure force is dominant. It was observed that the velocity profile creates a reverse flow, which leads to the sign change in the shear stress profile. When these two forces are equal and opposite, velocity and shear stress are zero.

Fig. 7(a) shows the influence of pressure and slip on the value of θ. When the pressure and electroosmotic forces are opposite or when the effect of the slip condition is strong, temperature decreases with a uniform slope from wall to center of the channel. However, when there is an adverse pressure gradient or a weak slip effect at the wall, the temperature profile shows a maximum value. In these cases, the viscous dissipation increases, driven by the rising velocity and subsequent increase in friction. The greatest viscous dissipation always occurs in the wall and the space around it because there is more friction in those areas. However, due to the constant temperature boundary condition at the wall, the heat generated internally by viscous dissipation accumulates near the wall, causing the temperature in the surrounding space to be higher than that of the wall. Therefore, a maximum point is observed.

**Fig. 7a:**
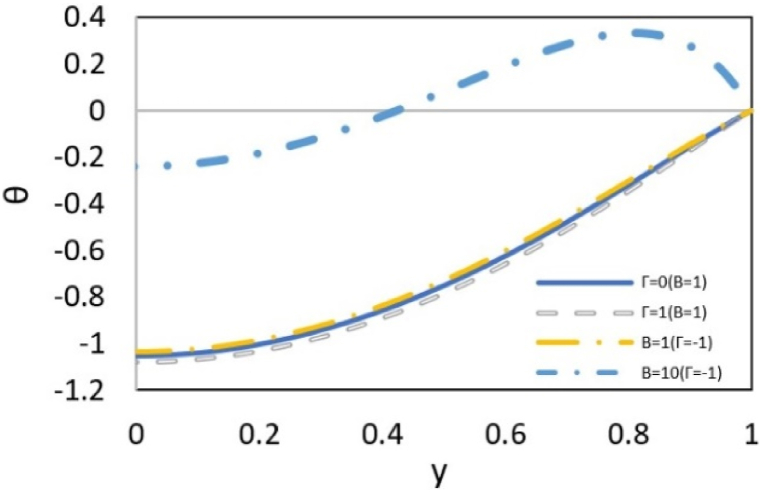
Effect of B and Γ on dimensionless temperature distribution at ${\varepsilon De}^{2}$ = 0.3, S = ζ = *Le* = Ec = 1, Pr = 5, Bc = Nb=Nt = 0.1, Re = 0–5, and k = 3.

Fig. 7(b)investigates the effect of the Eckert number (Ec) and the Prandtl number (Pr) on the dimensionless temperature profile. Since the Eckert number indicates the amount of viscous dissipation, an increase in this parameter signifies increased heat generation by viscous dissipation. As a result, by increasing the Eckert number, the local temperature in more areas of the channel space will be higher than the wall temperature. The Prandtl number shows the ratio of momentum diffusivity to thermal diffusivity. Therefore, increasing this parameter means higher momentum diffusivity or lower thermal diffusivity. Reinforcing momentum diffusivity leads to an increase in velocity gradient, subsequently resulting in increased viscous dissipation. Furthermore, the enhanced momentum diffusivity generates an inertia force that pushes nanoparticles toward the wall, causing an increase in particle collisions and higher heat generation near the wall. Decreasing thermal diffusivity increases the difference between wall and local temperatures. All these reasons create a maximum point in the graph by increasing the Prandtl number.

**Fig. 7b:**
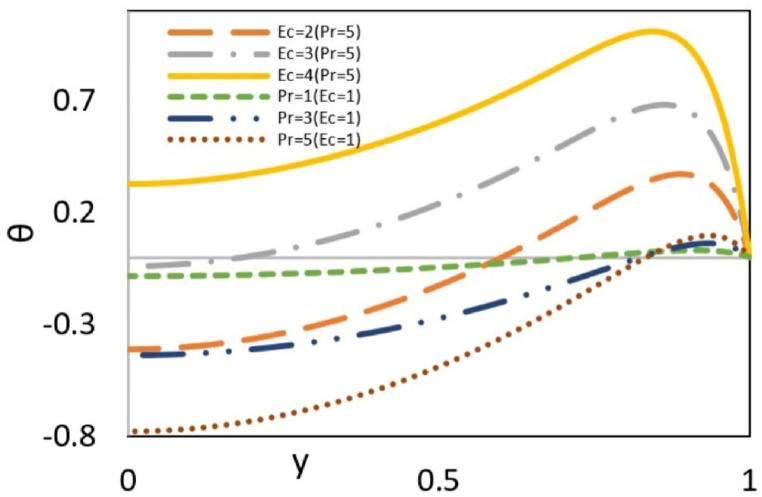
Effect of Pr and Ec on dimensionless temperature distribution at Γ = −1, ${\varepsilon De}^{2}$ = 0.3, S = ζ = *Le* = B = 1, Re = 0.5, Bc = Nb=Nt = 0.1 and k = 3.

The randomized motion of nanoparticles, driven by thermal effects and random forces, enhances mass transfer in nanofluid within the microchannel. This motion induces interactions between the liquid particles and the nanoparticles within the nanofluid due to the stochastic nature of particle movement. In microchannel, the stochastic movement of nanoparticles induces mass transfer, which denotes the migration of particles across regions with disparate concentration gradients within the nanofluid, thereby instigating concentration differences. The boundary condition specifies that the concentration remains uniform at the wall, denoted as a ${T=T}_{w}$ , throughout the process.

Fig. 8(a) examines the influence of Reynolds number (Re) on the distribution of nanoparticles (φ). The figure illustrates that an increase in the Reynolds number augments mass transfer. Additionally, as it approaches the center of the channel, the mass transfer profile exhibits a gentler slope. Increasing the Reynolds number means a reinforced inertial force in the fluid. The inertia is the force due to the momentum of the fluid, which is transferred from higher velocity in the center of the channel to lower velocity near the walls. This force is imposed on the nanoparticles and, as stated earlier, pushes them towards the wall. This leads to an increase in particle concentration at the wall and causes the concentrations to diverge gradually from c and $c_{w}$.

**Fig. 8a:**
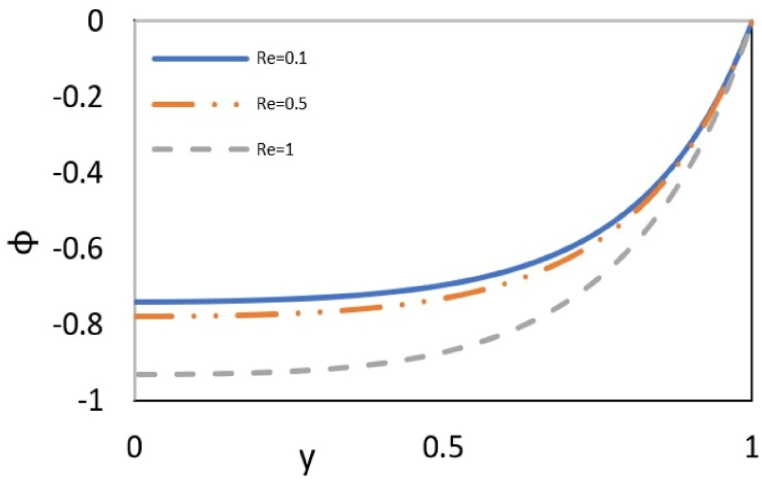
Effect of the Re on nanoparticles distribution at Γ = −1, ${\varepsilon De}^{2}$ = 0.3, S = ζ = *Le* = B = 1, Ec = Pr = 5, Bc = Nb=Nt = 0.1, k = 3.

Fig. 8(b)illustrates the effect of Lewis number on nanoparticles distribution, which shows the ratio of thermal diffusivity to mass diffusivity. An increase in the Lewis number weakens the nanoparticle's diffusion, which causes their accumulation near the wall and increases the concentration difference between c and $c_{w}$.

**Fig. 8b:**
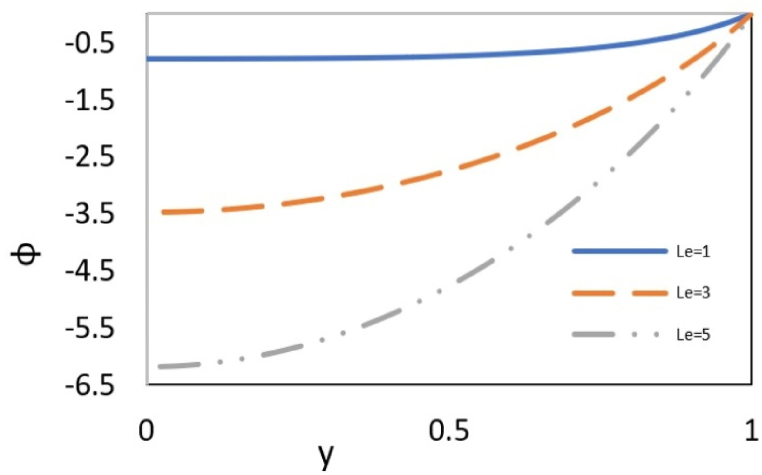
Effect of *Le* on nanoparticles distribution in a channel with Γ = −1, ${\varepsilon De}^{2}$ = 0.3, S = ζ = B = 1, Re = 0.5, Ec = Pr = 5, Bc = Nb=Nt = 0.1, and k = 3.

**Fig. 9:**
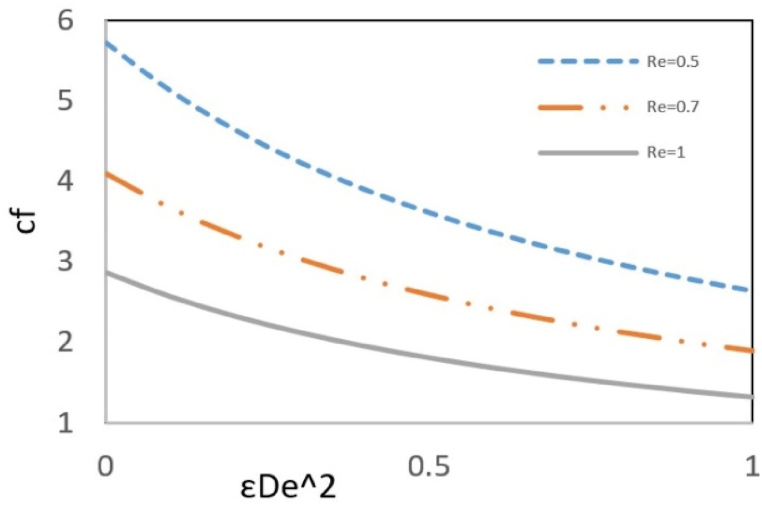
Effect of Re on Local skin friction factor versus ${\varepsilon De}^{2}$ at ζ = s = 1, k = 3, B = 10, Γ = y = −1 and Bc = 0.1.

In Fig. 9, the influence of Reynolds number and pressure gradient on the local friction coefficient at the wall, as a function of changes in the $\varepsilon De^{2}$, is investigated. The friction factor represents the ratio of the shear stress on the channel's bounding walls to the fluid dynamic pressure. As can be seen in the figure, the friction factor is reduced by elasticity; this reduction occurs because of the increasing $\varepsilon De^{2}$, due to the shear-thinning behavior of the fluid, decreases the shear stress at the wall, leading to a reduction in the friction factor. Also, results show that higher Reynolds numbers are associated with a decreased friction coefficient. This is caused by the dominant inertial forces, which increase the dynamic pressure and subsequently decrease the friction factor.

The local Nusselt number reflects the total heat exchange between a fluid and the microchannel wall to conductive heat transfer at a boundary in a fluid. Fig. 10(a) shows the effect of elasticity and Reynolds number on the local Nusselt number. Decreasing the Reynolds number and increasing elasticity generally increase the Nusselt number. The difference between wall and local temperatures is deriving force of heat transfer. The heat generated due to viscous dissipation increases local temperature while the temperature at the wall is constant due to the imposed boundary conditions. Therefore, the difference between these two temperatures and, subsequently, the heat transfer rate is reduced with increased viscous dissipation. An increase in the Reynolds number, due to the amplification of friction between the fluid and the wall, increases viscous dissipation and, as a result, decreases the Nusselt number. On the other hand, the growth of elasticity, because of the shear-thinning behavior of the fluid, reduces viscous dissipation, which causes the Nusselt number to increase. Additionally, as seen in Fig. 8(a(, an increase in the Reynolds number raises the nanoparticle concentration near the wall, which leads to more particle collisions that generate heat and subsequently reduce the Nusselt number. However, another notable point in the figure is related to Re = 1 and low elasticity, where the Nusselt number decreases until it approaches zero and then changes sign. The simultaneous effect of high Reynolds number and low elasticity leads to an intensive increase in viscous dissipation, which causes in the specific value of Reynolds number and elasticity, the local temperature near the wall will be equal to the wall temperature. This indicates no driving force for heat transfer, and the Nusselt number is zero. Increasing the Reynolds number or decreasing the elasticity of the fluid, more viscous dissipation occurs. This makes the local temperature near the wall higher than the wall temperature and changes the direction of heat transfer from the fluid to the wall. Therefore, the sign of the Nusselt number is positive. The influence of two Brownian motion and thermophoresis parameters are shown inFig. 10(b). Since both parameters are defined in terms of thermal diffusivity, their increasing effect is like reducing thermal conductivity. This implies that the contribution of conduction to the total heat transfer decreases, and as a result, the Nusselt number increases.

**Fig. 10a:**
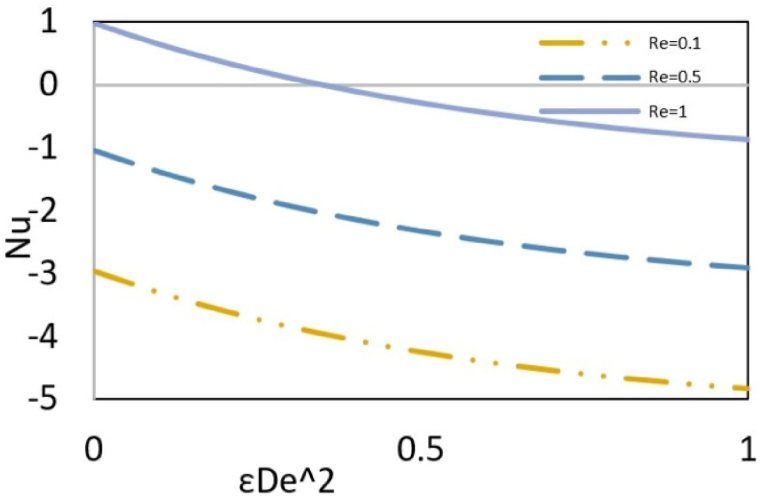
Effect of the Re on the local Nusselt number versus ${\varepsilon De}^{2}$ at Nt = Nb=Bc = 0.1, Pr = Ec = 5, ζ = B = s = *Le* = 1, k = 3, and y = Γ = −1.

**Fig. 10b:**
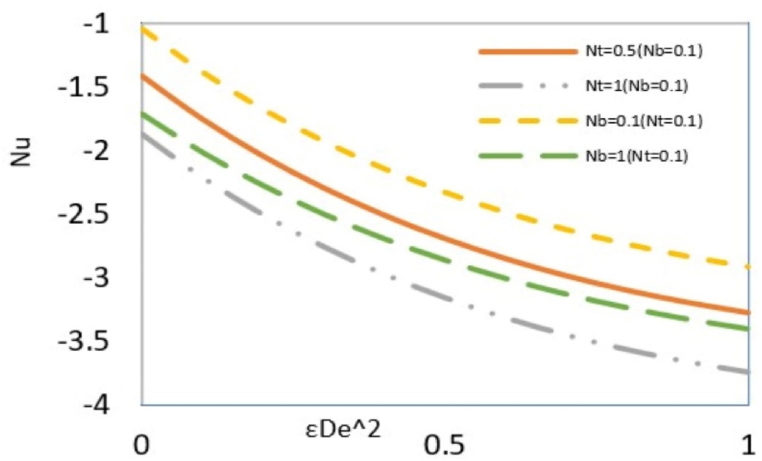
Effect of Nt and Nb on the local Nusselt number versus ${\varepsilon De}^{2}$ at Bc = 0.1, Ec = Pr = 5, ζ = B = *Le* = s = 1, k = 3, y = Γ = −1 and Re = 0.5.

The local Sherwood number signifies the ratio of convective mass transfer to diffusivity at a particular point within the fluid—an escalation in mass transfer through convection results in a corresponding increase in the local Sherwood number. According to Fig. 11(a), an increase in $\varepsilon De^{2}$ or the adverse pressure gradient increases the flow rate. As a result, the inertia force and momentum transfer push the nanoparticles toward the microchannel walls, which was discussed in the concentration distribution part. This migration of nanoparticles increases the difference between local and wall concentrations and, therefore, increases the Sherwood number. Also, an increase in the parameter k means decreasing the electric double-layer thickness, which causes a reduction in the build-up of ions at the boundary. Therefore, the difference between local and wall concentrations and, subsequently, the Sherwood number decreases.

**Fig. 11a:**
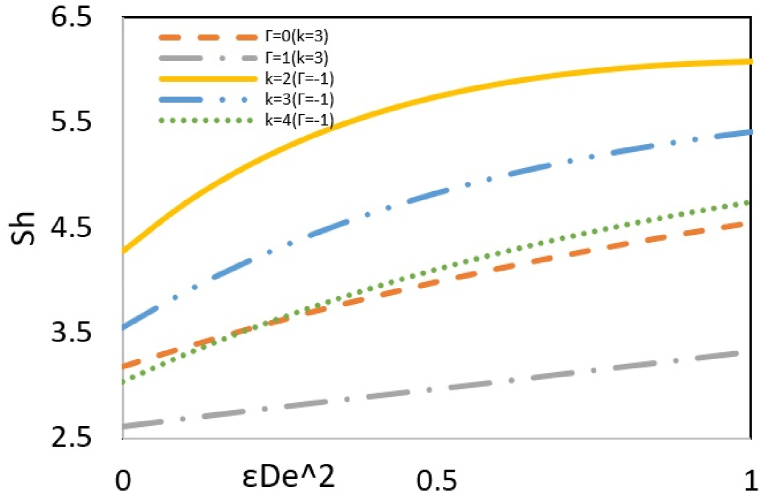
Effect of Γ and k on the local Sherwood number versus ${\varepsilon De}^{2}$ at Nt = Nb=Bc = 0.1, Pr = Ec = 5, ζ = B = s = *Le* = 1, y = −1 and Re = 0.5.

Fig. 11(b) investigates the effect of Brownian motion and thermophoresis parameters on the Sherwood number. Thermophoresis is a phenomenon observed in mixtures of mobile particles where the different particle types exhibit distinct responses to the force of a temperature gradient. The movement of particles from a cold to a hot region is called negative thermophoresis, typically occurring in lighter/smaller species. Since the nanoparticles are smaller than the base fluid particles, they tend to migrate toward the wall where the temperature is higher. Hence, the increasing difference between the local and wall concentrations leads to an enhancement of the Sherwood number. However, the Brownian motion parameter causes a decrease in the Sherwood number because increasing this parameter increases the contribution of diffusion relative to the total mass transfer, leading to a reduction in the Sherwood number.

**Fig. 11b:**
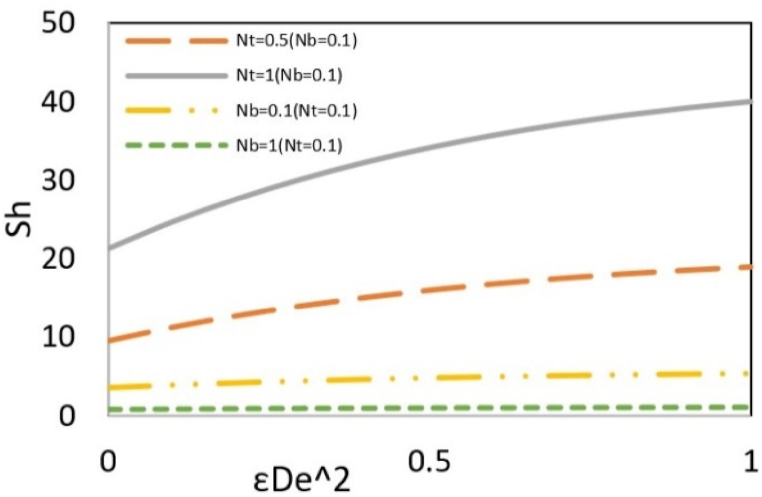
Effect of Nt and Nb on the local Sherwood number versus ${\varepsilon De}^{2}$ at Bc = 0.1, Ec = Pr = 5, ζ = B = *Le* = s = 1, k = 3, y = Γ = −1 and Re = 0.5.

## 4 Conclusion

The present study provided a comprehensive analysis of fluid flow characteristics and heat and mass transfer phenomena in nanofluid flow within a microchannel, influenced by both electroosmotic and pressure forces. The s-PTT viscoelastic model governed fluid rheology. The homotopy perturbation method presented an analytical solution for the constitutive equation of the problem. Results indicated that the elasticity of the fluid enhanced the flow rate, Nusselt number, and Sherwood number while reducing the fluid friction factor. However, the Reynolds number decreased both the Nusselt number and the friction factor. The Brownian motion and thermophoresis parameters increased the Nusselt number while they had two opposite effects on the Sherwood number. The thermophoresis parameter enhanced the Sherwood number, while the Brownian motion parameters reduced the Sherwood number. When the pressure and electroosmotic forces were applied in the same direction, they enhanced the flow rate and velocity; however, when applied in opposite directions, they could cause reverse flow. Additionally, a decrease in the double-layer thickness reduced the surface potential and increased the flow rate. Furthermore, an increment of slip at the walls increased the maximum velocity at the centerline of the channel. It also caused the disappearance of the maximum point in the temperature profile.

## CRediT authorship contribution statement

**Mahtiam Kananipour:** Writing – original draft, Validation, Software, Resources, Methodology, Investigation, Formal analysis, Data curation, Conceptualization. **Mehdi Moayed Mohseni:** Writing – original draft, Project administration, Methodology, Investigation, Funding acquisition, Conceptualization. **Reza Jahanmardi:** Validation, Software, Resources, Project administration, Methodology, Investigation, Funding acquisition, Formal analysis. **Hossein Ali Khonakdar:** Writing – review & editing, Visualization, Validation, Supervision, Software, Resources, Project administration, Funding acquisition, Conceptualization.

## Data availability

The data that support the findings of this study are available from the corresponding author upon reasonable request.

## Declaration of competing interest

The authors declare that they have no known competing financial interests or personal relationships that could have appeared to influence the work reported in this paper.
